# Medical and Sickness Benefits

**Published:** 1912-12-14

**Authors:** 


### OUR NATIONAL INSURANCE ACT DEPARTMENT.

### XXXVI.—Medical and Sickness Benefits.

In our last issue we dealt at length with the duties of Insurance Committees under the National Insurance Act, pointing out in particular that they are responsible for the administration of medical benefit both for members of approved societies and for deposit contributors. In order to avoid any possible confusion it would seem desirable clearly to define the difference between "medical benefit" and "sickness benefit."

Both benefits are defined in Section 8 of the Act, and it may, perhaps, serve a useful purpose if the precise words of the Act are used. "Medical benefit" is stated to be "medical treatment and attendance, including the provision of proper and sufficient medicines, and such medical and surgical appliances as may be prescribed by regulations to be made by the Insurance Commissioners."

"Sickness benefit" is defined as "periodical payments whilst rendered incapable of work by some specific disease or by bodily or mental disablement, of which notice has been given, commencing from the fourth day after being so rendered incapable of work, and continuing for a period not exceeding twenty-six weeks."

### Medical and Sickness Benefits Contrasted.

When set out side by side in this manner the differences are readily noted. Medical benefit is designed to meet the requirements of those insured persons who have need of medical treatment and attendance, and in many cases such necessity will arise when no claim could be substantiated for sickness benefit, for the latter can only be drawn when the illness is such as to render the insured person incapable of work. There is also the further qualification that the benefit commences as from the fourth day of incapacity, and consequently it may be stated generally that sickness benefit will only become payable in comparatively serious cases of illness, whereas medical benefit may be obtained in illnesses of much less serious consequence. It has to be borne in mind, also, that the objects of the benefits are essentially different. The object of providing free medical treatment and attendance is to prevent unnecessary delay in consulting a doctor on what may appear to be a minor ailment, and which so often is, in fact, of a trifling character at the moment, but may become by neglect a serious impairment of health. Thus medical benefit is intended to act not only as a curative for those who are in seriously bad health, but also as a preventive of illness by taking the case in hand in its early stages.

The fundamental object, on the other hand, of sickness benefit is to provide the means for maintaining the insured person and (or) his dependants when he (or she) is laid aside by some disablement which precludes the possibility of following the usual employment, and thus, presumably, of earning the wages on which his maintenance depends.

### Medical Benefit.

Since we went to press with our last issue three most important Memoranda dealing with the administration of medical benefit have been published. The first of these is a Government Memorandum setting forth the latest concessions made by the Chancellor of the Exchequer on behalf of the Government in regard to the administration of the benefit. The second is a Report issued by the British Medical Association upon the interviews between the Chancellor of the Exchequer and the committee of five appointed by the representative meeting of November 19 and 20 as to the conditions of service under the Act. The third is a Memorandum issued by the Insurance Commissioners summarising the result of the recent conferences with representatives of Insurance Committees, and setting forth the practical steps to be taken by the Committees in order to bring the provisions of the Act relating to medical benefit into operation on January 15 next.

It is evident, therefore, that matters are moving; and it may be remarked that every day is now of moment if the arrangements for the administration of the benefit are to be completed in time for the fulfilment of the pledge of the Government, supported by an Act of Parliament, to provide free medical treatment and attendance to insured persons after the expiration of six months from the commencement of the Act.

It may be remarked, in passing, that the waiting period of six months in the case of medical benefit only applies to the six months from the commencement of the Act, so that if a person were to enter insurance for the first time on January 15, and were to require the attendance of a doctor in the same week there is no waiting period at all, and the right to benefit would be immediate. In the case of sickness benefit the waiting period will always be six months from the entry into insurance, and may, in certain circumstances, be more; as, for instance, when there have been periods of unemployment and contributions have been allowed to fall into arrear, because there is the further qualification as to the right to benefit that at least twenty-six weekly contributions shall have been paid.

### The Government Memorandum.

The Government Memorandum deals principally with points that required elucidation in the Provisional Regulations issued by the Commissioners on October 1 last. The first of these points is of interest not only to the medical profession, but to insured persons generally, for the Government had been asked to define more clearly the duties of practitioners taking service under the Act. In the Provisional Regulations it had been laid down that the practitioner should give to all persons who were for the time being entitled to obtain treatment from him such treatment as is of a kind which can, consistently with the best interests of the patient, be properly undertaken by a practitioner of ordinary professional skill. Beyond emphasising the fact that the treatment contemplated did not involve specialised services, the Memorandum does not specifically define the duties, but the opinion is expressed that it will only be in rare instances that cases of doubt will arise. In these cases the practitioner, it is proposed, shall have the right of referring the matter to the local medical committee, and that that committee or the Insurance Committee should, if they think it desirable, refer the matter to a Court of Referees appointed for the purpose. The Court of Referees would be constituted by the Insurance Commissioners from among the medical and legal professions.

A large section of the Memorandum is concerned with the question of the remuneration of the doctors for extra services and mileage. It may be said, speaking generally, that in this matter the last word of the Government, in the offer of an extra £1,600,000 recently made, has been said. At the same time a small special fund is to be set up to meet the case of doctors with practices in very sparsely inhabited districts where attendance entails long and arduous journeys.

The other important matter in dispute was that of lay control. As we pointed out last week, the Act has given to the Insurance Committees a constitution which places the medical members in a minority, and in consequence practitioners taking service under the Act are, in effect, placing themselves under lay control. This cannot, apparently, be altered without an amending Act, which the Government is loth to introduce at the present time. The concessions offered are therefore in the nature of a palliative. It is proposed that complaints against doctors should first be considered by a Medical Service Committee, on which the medical profession and the insured should be equally represented. A complaint sifted by such a body in private session, before submission to the Insurance Committee, was thought to meet the views of the deputation and to ensure the protection of practitioners. In more serious cases, where the question at issue is the removal of a practitioner's name from the panel, the Government are prepared to issue regulations to the Insurance Committees requiring them to consider the recommendations of a Committee of Inquiry composed of medical men and one lawyer before coming to a decision.

### The Report of the British Medical Association.

The Report of the British Medical Association traverses much the same ground as the Government Memorandum to which we have just alluded, as indeed it had to. The Council of the Association does not offer any recommendation to the members, but, after a recital of the facts, leaves them to vote as they please on the question: "Are you in favour of the Association calling upon the profession to refuse to enter into any agreement with local Insurance Committees to give service under the Act upon the terms and conditions now finally offered by the Government?" The result of the voting is to be made known at a special representative meeting to be held on December 21. It is pointed out in the Report that, though the result of the voting will not be a binding decision of the Association, it must carry great moral weight.

In making this last statement the Association has apparently paved the way for a settlement of the dispute; for if the result of the voting means no more than that the minority who are in favour of accepting service are at liberty to do so, it would certainly seem that a sufficient, if not truly adequate staff of doctors will be found to be ready to enter into agreements with the Insurance Committees.

### The Memorandum of the Insurance Commissioners.

It is admitted by the Commissioners that the time is fast approaching for the completion of the arrangements; they therefore instruct the Insurance Committees, in the Memorandum alluded to, to issue to each practitioner upon the Medical Register in their respective areas a circular letter inviting him to complete a form of agreement for service under the Act. These letters are to be in the hands of the doctors by December 14, and the last day of the year is mentioned as the date by which acceptance must be notified. In the meantime, the doctors are requested to approach the Committees either individually or through any local association in regard to matters that are not beyond the scope of their powers of negotiation. The result of this invitation will be awaited with interest. We indicated last week that the Insurance Committees have already been supplied with the names and other particulars of insured persons residing in their respective areas for whom they are responsible in the matter of providing medical benefit. As some delay may arise at the outset in regard to the selection of a practitioner from the panel, and the acceptance by the doctor of the insured person as a patient, the approved societies have been furnished with vouchers having a currency up to April 30 next for distribution to their members. These are to be delivered by the societies to their members with the third quarter's insurance cards.

### Provision of Medicines and Appliances.

It will be noticed from the definition of medical benefit that provision is to be made for the supply of proper and sufficient medicines and certain prescribed medical and surgical appliances. These are not, however, to be supplied by the doctors, except in special circumstances, but are to be dispensed by chemists. The Act lays down elaborate provisions in the matter, by which, among other things, the Insurance Committees are required to prepare lists of duly qualified persons, firms, and bodies corporate who have agreed to supply the drugs, medicines, and appliances to insured persons.

The ordinary procedure will be for the doctor attending an insured person to furnish him with a ticket prescribing the medicine or appliance required, and this ticket, when presented to one of the chemists on the list, will entitle the insured person to the free supply of the article prescribed.

